# Effect of Basic Fibroblast Growth Factor on Orthodontic Tooth Movement in Rats

**Published:** 2013-08-24

**Authors:** Massoud Seifi, Mohammad Reza Badiee, Zahra Abdolazimi, Parisa Amdjadi

**Affiliations:** 1 Department of Orthodontics, Dental School, Shahid Beheshti University of Medical Sciences, Tehran, Iran; 2 Dentofacial Deformities Research Center, Dental School, Shahid Beheshti University of Medical Sciences, Tehran, Iran; 3 Dental School, Shahid Beheshti University of Medical Sciences, Tehran, Iran

**Keywords:** Orthodontic Tooth Movement, bFGF, Angiogenesis, Rat

## Abstract

**Objective:**

Basic fibroblast growth factor (bFGF) is a cytokine involved in angiogenesis, tissue remodeling and stimulation of osteoblasts and osteoclasts. The present study assesses the effects of a local injection of bFGF on the rate of orthodontic tooth movement.

**Materials and Methods:**

: In this laboratory animal study, we randomly divided 50 rats into 5 groups of 10 rats each. Rats received 0.02 cc injections of the following doses of bFGF: group A (10 ng), group B (100 ng) and group C (1000 ng). Group D (positive control) received an orthodontic force and injection of 0.02 cc phosphate buffered saline whereas group E (negative control) received only the anesthetic drug. A nickel titanium spring was bonded to the right maxillary first molar and incisor. After 21 days, the rats were sacrificed and the distance between the first and second right molars was measured by a leaf gauge with 0.05 mm accuracy. ANOVA and Tukey’s HSD statistical tests were used for data analysis.

**Results:**

The greatest mean value of orthodontic tooth movement was 0.7700 mm observed in group C followed by 0.6633 mm in group B, 0.5333 mm in group A, 0.2550 mm in group D and 0.0217 mm in group E. There was a significantly higher rate of tooth movement in the test groups compared to the control groups (p<0.05). Among the test groups, the rate of tooth movement in group C was significantly higher than group A (p<0.05). Weight changes after the intervention were not significant when compared to the baseline values, with the exception of group E (p>0.05).

**Conclusion:**

The effect of bFGF on the rate of tooth movement was dose-dependent. Injection of 1000 ng bFGF in rats showed the most efficacy.

## Introduction

The process of bone remodeling process usually involves resorption of old bone by osteoclasts and formation of new bone by osteoblasts. The amount of skeletal tissue depends on the equilibrium between the rate of new bone formation and old bone resorption during the process of physiologic growth and skeletal remodeling. Mechanical loads, in particular orthodontic forces, can also affect the bone remodeling process; but despite extensive investigations on animal models and human beings, its exact mechanism is yet to be fully understood (1-3). According to the classic theory of tooth movement, the tooth moves along the periodontal ligament (PDL) following the application of heavy orthodontic forces and some changes occur in the blood flow and oxygenation of the respective area that results in cell death and formation of an area of sterile necrosis. Compensatory hyperemia 

and secretions from the necrotic tissue cause accumulation of giant cells and phagocytes in the PDL which result in resorption of cement, bone and PDL (4). This stage of orthodontic movement is known as the lag phase of orthodontic treatment during which no tooth movement occurs. At present, there is a general consensus that not only the heavy loads, but also the light forces may cause sterile necrosis and delay tooth movement following application of orthodontic forces. The reason could be the structure of the PDL, its irregularities and bone spicules (5). Various studies have attempted to decrease the duration of lag phase of orthodontic treatments and increase the rate of tooth movement through enhancing the proliferation of osteoclasts and preventing the formation of sterile necrosis. Presently, biological molecules such as prostaglandins (PGs), vascular endothelial growth factor (VEGF) and basic fibroblast growth factor (bFGF) are increasingly used to expedite and facilitate orthodontic tooth movement in addition for the repair of periodontal furcation defects (6-8). In 2001 Kaku et al. (7) evaluated the effect of recombinant human VEGF (rhVEGF) on orthodontic tooth movement and demonstrated that this growth factor can enhance tooth movement through increasing the number of osteoclasts. Kohno et al. in 2003 found that during the application of mechanical orthodontic forces, expression of VEGF in osteoclasts at the pressure side increases as a paracrine factor. Also, they have shown that endogenous VEGF and injected rhVEGF expedited the amount of tooth movement (9). bFGF or fibroblast growth factors-2(FGF-2) is a polypeptide of the family of FGF which can be found in dentin. The role of this molecule is similar to that of VEGF and it is involved in immigration and proliferation of endothelial cells, angiogenesis under *in vivo* conditions and bone reconstruction (10, 11). This growth factor improves vascularization, enhances wound healing, regulates the bone mass and its formation, increases the number of osteoclasts, decreases the production of type I collagen and inhibits alkaline phosphatase activity (12).

During the course of treatment with fixed orthodontic appliances, patients’ esthetics and oral hygiene are compromised. Therefore, shortening the treatment course as much as possible will greatly enhance patient satisfaction and decrease inevitable complications of orthodontic treatment. On the other hand, blood vessels present in the PDL play a pivotal role in the tissue remodeling process. If we increase angiogenesis in this area by using bFGF, we may be able to reduce the lag phase in orthodontic treatments and subsequently facilitate tooth movement.

The present study sought to assess the effect of different doses of bFGF on orthodontic tooth movement in rats.

## Materials and Methods

**Fig 1:**
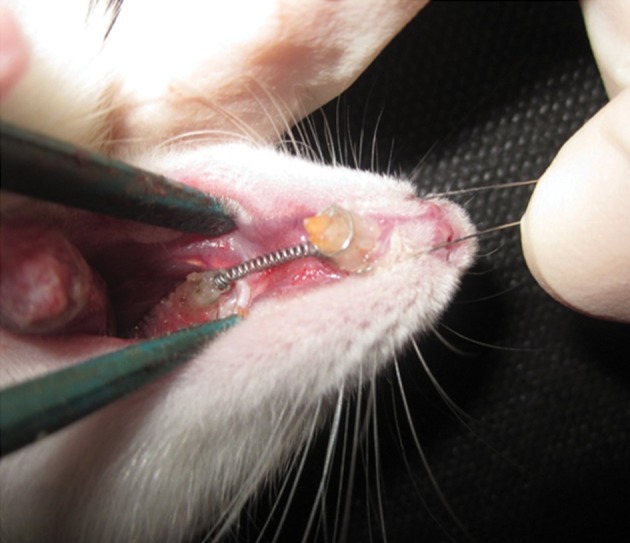
Placement of the orthodontic appliance.

**Fig 2:**
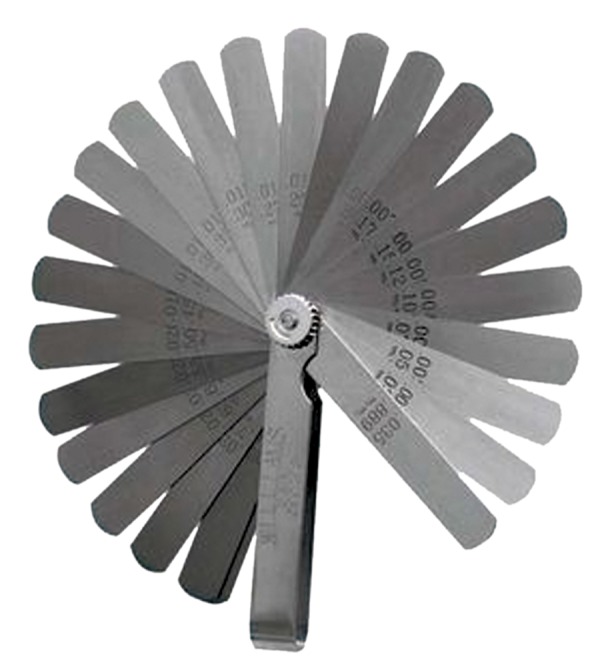
Leaf gauge.

zed single blind laboratory animal study. We collected data through observation, information form completion and the use of specific tables. Manipulation and treatment of the animals was performed according to the approved protocol of the Institutional Animal Care and Usage Committee and the approval of the Ethical Committee of the Shahid Beheshti University of Medical Sciences Dental School. Nonrandomized convenience sampling was performed to select study subjects among those who met the inclusion criteria. Selected samples were divided into five groups of two controls and three tests using the simple random sampling method. A total of 50 male Wistar rats (SCL, Shizuoka, Japan) with a mean age of 4 months and mean weight of 330 ± 30 g were kept in the animal room of Shahid Beheshti Dental School under similar light and nutritional conditions for two weeks to become acquainted with the new environment. Rats were randomly divided into five groups of ten each. Each group (n=10) of rats was dyed and kept in separately numbered cages. The rats in group D (positive control) each received an injection of 0.02 cc phosphate buffered saline and an orthodontic appliance. The ten rats in group E (negative control) did not receive the orthodontic appliance or bFGF. During the study period, this group only received anesthetic drug. The remaining 30 rats were randomly assigned to three groups, A which received 0.02 cc of 10 ng bFGF (Royan Institute), B (0.02 cc of 100 ng bFGF) and C (0.02 cc of 1000 ng bFGF). At the beginning of the study we weighed each rat on a digital scale (Shimadzu, Kyoto, Japan, 61189). Rats were anesthetized by intraperitoneal injections of 20 mg/kg ketamine hydrochloride (Rotexmedica, Trittau, Germany) and 2 mg/kg xylazine (Bayer, Levekysen, Germany) administered with an insulin syringe. Post-anesthesia care included monitoring of vital signs, maintaining adequate room temperature, and rotating the rats from side to side once every few minutes in order to prevent pulmonary edema. A NiTi closed coil spring (Ormco® , Orange, USA) was ligated between the right permanent maxillary first molar and the right central incisor using a stainless steel ligature wire (0.01 inch, 3 M, Unitek, Monrovia, CA, USA) and fixed with a self-cure composite resin [3 M, Unitek, Monrovia, USA) Fig 1]. We applied a 60 g force, which was measured by a force meter. The type of tooth movement induced was tipping. An insulin syringe was used to inject a total of 0.02 cc of the appropriate dose of bFGF into the buccal vestibular mucosa next to the mesial root of the first molar. During the study period, a soft diet was provided for all groups. After 21 days, rats were placed in a desiccator that contained saturated chloroform and sacrificed through inhalation. They were then decapitated and prior to removal of the orthodontic appliance the distance between the first and second right molars was measured by a leaf gauge with 0.05 mm accuracy (Fig 2). The measurements were repeated three times by the same technician blinded to the study using the same tool. The mean of the three measurements was reported as the final value.

## Results

The highest mean value of orthodontic tooth movement was 0.7700 mm which was observed in group C, followed by 0.6633 mm in group B, 0.5333 mm in group A, 0.2550 mm in group D and 0.0217 mm in group E (Table 1). Paired sample test found a statistically significant difference between the three test groups and the control group with the orthodontic appliance (D) in terms of tooth movement (p<0.05). However, this difference for the control group without the orthodontic appliance (E) was not statistically significant (p>0.05). ANOVA also showed a significant difference in the mean value of tooth movement between different groups (F=30.054, p<0.05). 

**Fig 3:**
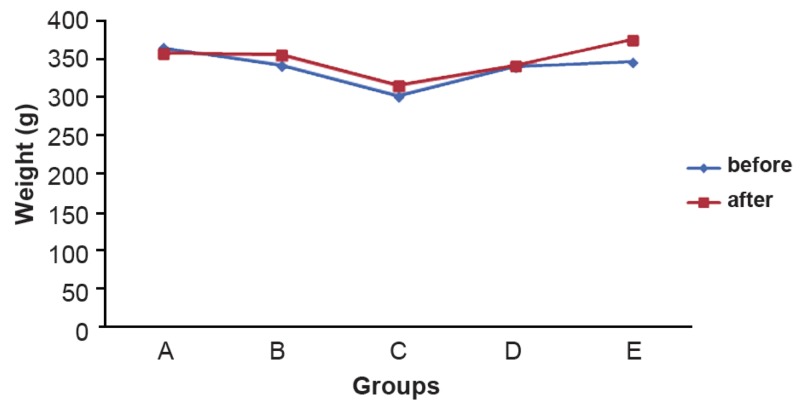
The mean weight at the beginning and end of the study in the different groups.

Tukey’s HSD analysis was used for comparison of the mean tooth movement between different groups. Based on this analysis, we detected a significant difference in the mean tooth movement between the three test groups (A, B and C) and the two control groups (D and E; P<0.05). There was a higher rate of tooth movement in the test groups than in the controls. Also, the rate of tooth movement differed between the test groups as this movement was significantly greater in group C compared to group A (p<0.05). Additionally, significant differences existed between the control group with the orthodontic appliance (group D) and the control group without the appliance (group E) in terms of mean tooth movement (p<0.05). This movement was greater in group D rats. Although the tooth movement in group C was greater than in group B, and group B was greater than group A, this difference was not statistically significant (p>0.05; Table 1). In this study, we weighed all rats at the beginning and end of the study. According to the paired sample test, the weights of all animals at the end of the study increased compared to the baseline values for all groups, with the exception of group A. The weight change in groups A, B, C and D was not significant (p>0.05), however group E showed a statistically significant increase in weight (p<0.05, Fig 3).

ANOVA showed a significant difference in weight change between various groups (F=4.668, p<0.05). Tamhane analysis was used for comparison of weight change between different groups. This analysis indicated that the difference in weight change between groups E and A was statistically significant (p<0.05, Fig 3).

**Table 1:** The mean, standard deviation and range of maxillary right first molar movement in different study groups


	A	B	C	D	E

**Mean**	0.5333**	0.6633	0.77	0.2550*	0.0217*
**SD**	0.177	0.226	0.249	0.104	0.324
**Range**	0.3-0.82	0.32-0.93	0.47-1.22	0.12-0.48	0.0-0.08


Values are expressed as mean ± SD (n=10). * ; Significant differences as compared with the control group at p<0.05 and **; Significant differences as compared with the Cs group at p<0.05.

## Discussion

The present study evaluated the effect of bFGF on rate of tooth movement in 50 rats equally divided into three test and two control groups. Different doses of this material were injected into the buccal mucosa adjacent to the mesial root of maxillary right first molar

bFGF or FGF-2 is a cytokine that belongs to the FGF super family and is present in many tissues. This material is known as a growth factor that has multiple effects on cells and tissues. Most of its actions are attributed to its 18 kD isoform. At first, bFGF was thought to be a fibroblast mitogenic substance but further studies have shown its other activities such as angiogenesis stimulation, induction of endothelial cell proliferation, involvement in tissue remodeling, enhancement of wound healing, stimulation of proliferation and differentiation of osteoblasts and osteoclasts, chemotaxis of macrophages, involvement in fetal development, and protection of nerve cells against apoptosis (13-16).

onducted by Miyagawa et al. (17) it was shown that following application of a compressive force, an area of sterile necrosis developed one to seven days later. During the first week after initiation of tooth movement, expression of VEGF increased in PDL cells adjacent to the hyalinized area and the alveolar bone on the compressive side. In order to answer the question whether the compressive force directly induced the expression of VEGF or it was due to the vascular changes and decreased oxygenation, the same authors performed an* in vitro* study on human PDL cells. They have determined that an optimal force is required for expression of VEGF. Forces above this optimum value will decrease VEGF expression. According to their study, the VEGF produced in the pressure side plays an important role in angiogenesis although other angiogenic factors such as FGF-2, TNF-α and TGF-β may be involved. Zhang et al. (18) evaluated the impact of 10, 50 and 100 g orthodontic forces on expression of bFGF in rabbit’s periodontium. According to immunohistochemistry analyses, they detected a statistically significant difference only between the test group under 50 g forces and the control group. They have stated that a sensible force was required for expression of bFGF. In a study by Tang and Liu in 2005 that evaluated bFGF expression in periodontium of young rats during orthodontic treatment, it was revealed that expression of this factor in fibroblasts and osteoblasts of the test group at days 10 and 14 was higher than in the control group (19).

The present study demonstrated that the rate of tooth movement significantly increased in all groups who received bFGF injections compared to the control groups. The greatest mean value of tooth movement was 0.7700 mm which was observed in the group that received 1000 ng of bFGF (group C), which was significantly higher than the rate observed in control group D that had an orthodontic appliance (0.2550 mm). QI et al. in 2009 assessed the effect of recombinant mouse bFGF (rmgFGF) injections in rats with periodontitis during orthodontic treatment. They have stated that extrinsic, injected bFGF plays a key role in PDL remodeling during orthodontic movement (20). In another study by Wu and Liu the influence of a combined application of bFGF and insulin-like growth factor (IGF-1) on orthodontic tooth movement at days 1, 3, 7, 14 and 21 post-intervention was evaluated. They reported a significantly greater rate of tooth movement at days 7, 14 and 21 in the test group compared to the control group (21). Based on the Lin et al. study, FGF-8 has been shown to play a key role in normal bone metabolism because this growth factor is produced in the bone microenvironment. They have suggested a possible effect of this cytokine on bone remodeling by uncoupling the actions of osteoclasts and osteoblasts (22). Manabe et al. have reported that induction of osteoclastogenesis by an increase in endogenous FGF-2 production in the synovial fluid may result in joint destruction in rheumatoid arthritis patients (23). Kohno and coworkers have assessed the role of rhVEGF, another member of the growth factor’s family, in the amount of experimental tooth movement in mice. They showed that no significant differences existed between the cases and controls until day 15, however the difference between the two groups from days 18 to 21 was statistically significant. The reason for this difference was thought to be the lack of osteoclastic induction and subsequent root resorption in the rhVEGF group until day 14 (9). In another study the same researchers evaluated the effect of polyclonal antiVEGF antibody on the number of osteoclasts, amount of tooth movement and the degree of tooth relapse. They concluded that injection of this material during tooth movement resulted in a marked reduction in the number of osteoclasts, inhibition of tooth movement and relapse of teeth that moved (24). Kaku et al. (7) studied the effects of rhVEGF on the rate of osteoclast induction by testing various doses (0.1, 0.5, 1.0 and 1.5 µg). The results indicated that an injection of at least 0.5 µg of this material obtained the highest number of osteoclasts.

The role of bFGF in angiogenesis, osteogenesis, fibroblast induction and macrophage induction has been well demonstrated in various *in vivo* and* in vitro* studies (13-16, 25). One reason for the decreased rate of tooth movement after the initial phase is the discontinuation of blood flow and reduced oxygenation at the pressure side which leads to the development of sterile necrosis. Blood vessels present in the PDL are responsible for oxygenation and cellular nutrition which are the necessary elements for all cellular activities in health and disease conditions. Therefore, they are important factors for the tissue remodeling process following orthodontic tooth movement. As mentioned earlier, discontinuation of blood flow, formation of necrosis and subsequent deceleration of tooth movement following the application of light and heavy forces are inevitable. Thus, if the formation of a necrotic area can be prevented through the manipulation of one of the factors involved in angiogenesis or even decrease its duration and presence in PDL, we can expect more rapid tooth movement following reduction or elimination of the lag phase.

 phase is expected to reduce or vanish through increased angiogenesis and subsequent oxygenation and by induction of macrophages and secondary messengers involved in tooth movement. On the other hand, the proliferation and differentiation ability of various cells such as osteoblasts and osteoclasts and their important role in PDL remodeling during orthodontic therapy will also result in increased tooth movement (10, 16). This theory may explain the increased tooth movement observed in the present study following the administration of bFGF.

In the current study, the rate of tooth movement differed between the groups following administration of various doses of bFGF. In group C (1000 ng) we observed a significantly greater rate of movement than in group A (10 ng) which showed the dose-dependent nature of bFGF. Thus, we can state that for maximum tooth movement in rats, a dose of 1000 ng bFGF is required. 

Willems et al. studied the effect of microspheres that contained 10.5 µg of bFGF, VEGF or both, that were placed within an allograft transplanted in a rat model on longterm angiogenesis and osteogenesis. There was a significantly higher rate of angiogenesis and bone remodeling in the VEGF group at 4 and 18 weeks post-intervention than in the control group. bFGF did not cause a strong angiogenic or osteogenic response. A synergistic effect between these two growth factors was not observed. According to these researchers, an explanation could be the limited functional threshold of bFGF. Over-expression and longterm application of bFGF can result in apoptosis of osteoblasts. Therefore, the functional range of bFGF is highly dose-dependent (15). The results of the Willems et al. study have contrasted our findings. This discrepancy may be attributed to their longer study period and use of higher doses of bFGF in the form of a microsphere, which results in continuous and localized delivery of this growth factor to the respective site.

Our study duration was 21 days as required for induction of osteoclasts according to studies by Wu and Liu (21) and Kohno et al. (9); whereas, samples were evaluated at 4 and 18 weeks in Willems’ study. On the other hand, various researchers have stated that despite the role of bFGF in differentiation, maturation and apoptosis, the exact interactions of this growth factor have yet to be clearly understood. It seems that the maturation of osteoblasts is more susceptible to the change in dose of bFGF compared to other factors such as VEGF. Long-term exposure to bFGF results in apoptosis of mature osteoblasts and therefore, this growth factor has a limited spectrum of therapeutic efficacy (26-28).

In the present study, rats were weighed at the beginning and end of the study. Weight gain at the end of study in groups B, C and D and weight loss in group A were not statistically significant. However, there was a statistically significant weight gain in group E (p<0.05). The alterations in weight could be due to interference by the orthodontic appliance with the animals’ nutrition because orthodontic treatment in humans also changes the nutritional regimen. The changes in weight observed in our study agreed with the findings of Kale et al. who did not witness any weight loss in their study on rats (29). However, Roche, in his study on rabbits mentioned weight gain in all animals in the control group at the end of the 21-day study period. In the test group, all subjects gained weight at the end of study with the exception of two cases that lost weight (30). In contrast to a study by Akin et al. (31) that showed a significant reduction in weight at day 3 in all groups compared to control group, we found no significant differences in weight changes among groups except for that observed in group E. 

## Conclusion

Injection of bFGF can significantly enhance tooth movement. Its effect is dose-dependent and injection of 1000 ng in rats has the greatest efficacy. The present study results have indicated that local application of angiogenic factors such as bFGF can reduce the duration of orthodontic treatment. However, further studies are required on this subject.

## References

[1] Fujimura Y, Kitaura H, Yoshimatsu M, Eguchi T, Kohara H, Morita Y (2009). Influence of bisphosphonates on orthodontic tooth movement in mice. Eur J Orthod.

[2] Gonzales C, Hotokezaka H, Matsuo K, Shibazaki T, Yozgatian JH, Darendeliler MA (2009). Effect of steroidal and nonsteroidal drugs on tooth movement and root resorption in the rat molar. Angle Orthod.

[3] Proffit WR, Fields HW, Sarver DM (2007). Contemporary orthodontics.

[4] Wise GE, King GJ (2008). Mechanisms of tooth eruption and orthodontic tooth movement. J Dent Res.

[5] von Bohl M, Maltha JC, Von Den Hoff JW, KuijpersJagtman AM (2004). Focal hyalinization during experimental tooth movement in beagle dogs. Am J Orthod Dentofacial Orthop.

[6] Seifi M, Eslami B, Saffar AS (2003). The effect of prostaglandin E2 and calcium gluconate on orthodontic tooth movement and root resorption in rats. Eur J Orthod.

[7] Kaku M, Kohno S, Kawata T, Fujita T, Tokimasa C, Tsutsui T (2001). Effects of vascular endothelial growth factor on osteoclast induction during tooth movement in mice. J Dent Res.

[8] Murakami S, Takayama S, Kitamura M, Shimabukuro Y, Yanagi K, Ikezawa K (2003). Recombinant human basic fibroblast growth factor ( bFGF) stimulates periodontal regeneration in class II furcation defects created in beagle dogs. J Periodontal Res.

[9] Kohno S, Kaku M, Tsutsui K, Motokawa M, Ohtani J, Tenjo K (2003). Expression of vascular endothelial growth factor and the effects on bone remodeling during experimental tooth movement. J Dent Res.

[10] Qu D, Li J, Li Y, Gao Y, Zuo Y, Hsu Y (2011). Angiogenesis and osteogenesis enhanced by bFGF ex vivo gene therapy for bone tissue engineering in reconstruction of calvarial defects. J Biomed Mater Res A.

[11] Derringer KA, Linden RW (2004). Vascular endothelial growth factor, fibroblast growth factor 2, platelet derived growth factor and transforming growth factor beta released in human dental pulp following orthodontic force. Arch Oral Biol.

[12] Sako E, Hosomichi J (2010). Alteration of bFGF expression with growth and age in rat molar periodontal ligament. Angle Orthod.

[13] Depprich RA, Meyer U, Meyer T, Handschel J, Wiesmann HP (2009). Biomolecule use in tissue engineering. Fundamentals of tissue engineering and regenerative medicine.

[14] Okada-Ban M, Thiery JP, Jouanneau J (2000). Fibroblast growth factor-2. Int J Biochem Cell Biol.

[15] Willems WF, Larsen M, Friedrich PF, Shogren KL, Bishop AT (2012). Induction of angiogenesis and osteogenesis in surgically revascularized frozen bone allografts by sustained delivery of FGF-2 and VEGF. J Orthop Res.

[16] Feito MJ, Lozano RM, Alcaide M, Ramirez-Santillan C, Arcos D, Vallet-Regi M (2011). Immobilization and bioactivity evaluation of FGF-1 and FGF-2 on powdered silicon-doped hydroxyapatite and their scaffolds for bone tissue engineering. J Mater Sci Mater Med.

[17] Miyagawa A, Chiba M, Hayashi H, Igarashi K (2009). Compressive force induces VEGF production in periodontal tissues. J Dent Res.

[18] Zhang L, Zhuge CG, Mei YS, Zhang J (2010). Expressions of bFGF in rabbits’ periodontium under different orthodontic forces. Journal of Shandong University (Health Sciences).

[19] Tang XB, Liu J (2005). Changes of basic fibroblast growth factor in periodontal tissue during orthodontic tooth movement in young rat. Journal of Jinan University of Natural Science and Medicine.

[20] Lu-lu Q, Ying Z, Peng-jun L, Jian-Hua F, Shu-yan L (2009). Effect of recombinant mouse fibroblast growth factor-basic on tooth movement during orthodontic treatment in rats with periodontitis. Acta Academiae Medicinae CPAF.

[21] Wu Lp, Liu Tt (2009). Influence of combinative application of bFGF and IGF-1 upon periodontium remodeling in orthodontic tooth movement in rats. Heilongjiang Medicine And Pharmacy.

[22] Lin JM, Callon KE, Lin JS, Watson M, Empson V, Tong PC (2009). Actions of fibroblast growth factor-8 in bone cells* in vitro*. Am J Physiol Endocrinol Metab.

[23] Manabe N, Oda H, Nakamura K, Kuga Y, Uchida S, Kawaguchi H (1999). Involvement of fibroblast growth factor-2 in joint destruction of rheumatoid arthritis patients. Rheumatology (Oxford).

[24] Kohno S, Kaku M, Kawata T, Fujita T, Tsutsui K, Ohtani J (2005). Neutralizing effects of an anti-vascular endothelial growth factor antibody on tooth movement. Angle Orthod.

[25] Montesano R, Vassalli JD, Baird A, Guillemin R, Orci L (1986). Basic fibroblast growth factor induces angiogenesis* in vitro*. Proc Natl Acad Sci USA.

[26] Mayr-Wohlfart U, Waltenberger J, Hausser H, Kessler S, Gunther KP, Dehio C (2002). Vascular endothelial growth factor stimulates chemotactic migration of primary human osteoblasts. Bone.

[27] Marie PJ (2003). Fibroblast growth factor signaling controlling osteoblast differentiation. Gene.

[28] Mansukhani A, Bellosta P, Sahni M, Basilico C (2000). Signaling by fibroblast growth factors (FGF) and fibroblast growth factor receptor 2 (FGFR2)-activating mutations blocks mineralization and induces apoptosis in osteoblasts. J Cell Biol.

[29] Kale S, Kocadereli I, Atilla P, Aşan E (2004). Comparison of the effects of 1, 25 dihydroxycholecalciferol and prostaglandin E2 on orthodontic tooth movement. Am J Orthod Dentofacial Orthop.

[30] Roche JJ, Cisneros GJ, Acs G (1997). The effect of acetaminophen on tooth movement in rabbits. Angle Orthod.

[31] Akin E, Gurton AU, Olmez H (2004). Effects of nitric oxide in orthodontic tooth movement in rats. Am J Orthod Dentofacial Orthop.

